# Supplementary material for: Defining the determinants of protection against SARS-CoV-2 infection and viral control in a dose-down Ad26.CoV2.S vaccine study in nonhuman primates
Source: PLoS Biol. 2022 May 5;20(5):e3001609. doi: 10.1371/journal.pbio.3001609 (PMC9071142; doi:10.1371/journal.pbio.3001609)
Supplement: Attachment — Submitted filename: Responses to reviewer comments.docx [file pbio.3001609.s004.docx]

Dear Paula Jauregui,

We would like to thank the editor and the reviewers for their constructive comments on our paper, that collectively stated that our paper furthers our knowledge on the vaccine-mediated correlates of protection against SARS-CoV-2. We agree that further clarification and editing was needed to fully articulate the integrated nature of the multi-factorial immune correlates of protection against SARS-CoV-2. To address the reviewer’s comments we have now:

- added further statistical analysis to figure 6 and additional explanation of the statistical analysis to the figure legend.
- altered the text in line 106-109, and 122-126 has been changed to reflect the results of the statistical analysis more closely
- added a link to the data may be found, <https://github.com/dzhu8/Ad26-Dose-Down/>
- added further method details to the ELISPOT assay in Materials and Methods
- modified the text in line 104-105 to clarify our data comes from 6 weeks post vaccination and prior to challenge
- added additional text in line 339 and 348 highlighting that these conclusions are based in the context of Ad26 vaccines and need further testing to confirm for other vaccine platforms
- modified (increased) the font size to increase readability of various graphs
- added a new figure 5 to demonstrate the relative contribution of FcγR binding antibodies to each domain of spike towards the overall binding profile of spike.
- corrected all typos or omissions have been corrected in the figure legends in line 101, line 154-155, line 162-163
- added a paragraph to the discussion on the relevance of ADCC versus other humoral mechanisms of control of SARS-CoV2 given the limited evidence of Spike expression on the surface of cells
- added a paragraph to highlight the relevance of these findings to the current Omicron wave.

The unique opportunity to perform deep immunological investigation on this dose-down vaccine study, linking neutralizing, cellular, and Fc-effector function, provided an opportunity to examine the contribution of each immune mechanism in antiviral immunity to SARS-CoV2. We believe that the modifications create a balanced and rigorous interpretation of the data, providing new insights on immune correlates of protection against transmission and disease attenuation. These data are of considerable interest given the remarkable spread of Omicron, that is highly transmissible, but milder in individuals with previous immunity, highlight the critical importance of non-neutralizing humoral and cellular immunity against disease.

Once again, we thank the editor and the reviewers for their insightful comments and assistance in improving the manuscript.  Please find our point-by-point response to the reviewer’s comments attached.

Sincerely,

Galit Alter

**Defining the determinants of protection against SARS-CoV2 infection and viral control in a dose down Ad26.CoV2.S vaccine study in non-human primates- Reviewer Responses**

Daniel Zhu, Matthew Gorman

January 9^th^, 2021

We thank the reviewers for their critical assessment of our work. In the following, we address their concerns point-by-point.

**Reviewer 1**

This is beautiful study that attempts to discern the correlates of protection for SARS-CoV-2 vaccination. Through detailed analyses the authors link neutralizing antibodies to protection from infection and T-cell responses to control of viremia in infected animals. They provide evidence that Fc-dependent antibody functions complement neutralizing antibody responses and T-cell responses to confer protection and viral control, respectively. I have minor questions/comments for the authors.

The authors should make an attempt to address where the Fc functions are coming from. Are these functions mediated by the neutralizing antibodies, or are they originating from non-neutralizing antibodies? Is there any way to absorb neutralizing antibodies and assess the importance of the Fc functions mediated by non-neutralizing antibodies? This would be very informative regarding the value of binding non-neutralizing antibodies for protective outcomes against SARS-CoV-2 exposure and infection.

- **Response:** We agree with the editor and reviewer that understanding the contribution of neutralizing and non-neutralizing antibodies to the Fc-effectors is important. However, there is no currently no technique for directly isolating the neutralizing and non-neutralizing antibodies from a serum sample. We have tried many different strategies to address purification approaches to attempt to disentangle the biology of neutralizing and non-neutralizing antibodies, but have not been able to separate this biology- due to the fact that antibodies that neutralize likely often also mediate Fc-effector functions. However, to address the reviewer’s question – we have attempted to address this point by examining epitope specific Fc-profiles. Specifically, since most neutralizing antibodies target the receptor binding domain (RBD) of spike (PMID: 32454513), examination of RBD-specific and non-RBD-specific antibody profiles may provide some information towards addressing this question. Our Luminex experiment examined the ability of antibodies binding Spike, S1, S2, RBD, and NTD to bind Fcγ receptor. Comparison of RBD versus NTD and S2, which are targeted by fewer neutralizing antibodies, demonstrated that RBD epitope-targeting antibodies and non-RBD epitope-targeting antibodies follow similar Fc-receptor binding profiles. In fact, although fewer NTD-specific antibodies were observed compared to RBD- and S2-specific antibodies, the ability of RBD-, NTD-, and S2- specific antibodies to bind to Fc-receptors follow similar dose responses, pointing to parallel evolution of Fc-effector function across the Spike epitopes. Interesting differences were noted in the magnitude of the binding of NTD-specific antibodies across Fc-receptors, with dampened binding to FcγRIIa-3 compared to other Fc-receptors pointing to additional differences in epitope-specific evolution (**figure 5A** and attached below).
- Additionally, inspired by this approach to addressing the role of neutralizing/non-neutralizing antibodies in protection, we next used these data in a partial least squares regression model to determine the potential differential contribution of RBD-targeting, S2-targeting and NTD-targeting antibodies to protection, regressing on the area-under-the-curve of the viral load as measured by bronchoalveolar lavage for the entire 10-day timecourse (**figure 5B** and attached below). The enrichment of both RBD-targeting and S2-targeting features along the first latent dimension towards the protected animals suggests that both RBD-specific or non-RBD-specific antibodies likely contribute to protection via elicitation of Fc effector functions. We have added this figure and points to the manuscript.

Figure 5:

The authors did not complete the query about where data may be found. Currently, this box contains "XXX".

- **Response:** We apologize for this omission, and have how added the link to the data, which can be retried in a Github repository: <https://github.com/dzhu8/Ad26-Dose-Down/>.

Line 101 in the legend for Figure 1 - "relates" should be "relate".

- **Response:** We thank the reviewer for noting this error. The figure caption in question has been edited.

For the data in Figure 2, the authors make the claim that the lower two doses of the vaccine induce significantly lower responses. However, as presented in the Figure, significance is only apparent for the lowest dose for most variables. The text should be amended to more closely reflect the data as presented.

- **Response:** We appreciate the reviewer’s comment and agree that while there is a trend, significance only pertains to the final dose. We have therefore modified the text on lines 106-107 to clearly reflect this point, amending “slightly lower levels at the two lower doses” to “slightly lower levels at dose III and significantly lower levels at dose IV”. Line 108 was amended from “more significant loss of FcR binding was observed across the other vaccine dose groups” to “more significant loss of FcR binding was observed in dose group IV”. Lines 122-126 have now been amended to read “Similar to FcR binding profiles, similar antibody effector functions were observed in the top 2 dose-groups (**Fig 2I-L**), with a significant decline in dependent monocyte phagocytosis (ADCP) and antibody dependent neutrophil phagocytosis (ADNP) in group III and significant decline in ADCP, ADNP, antibody dependent complement deposition (ADCD), and antibody mediated NK cell activating (ADNKA) activity in group IV”.

Legend for Figure 3 - the colors listed do not correspond to the colors used in the actual figure.

- **Response:** We apologize for this confusion and have now modified the figure 3 caption in two places (lines 150-151 as well as lines 158-159) to indicate the correct color codes used in the figure.

Figure 6 - no statistical analyses are shown.

- **Response:** We thank the reviewer for noting this error and have added the statistical analysis in figure 6. We have also included the analysis information in the legend: “A two-way ANOVA with Tukey correction for multiple comparisons was used to compare antibody levels between groups. Only significant comparisons are shown. ns, not significant, *p≤0.05, **p≤0.01, ***p≤0.001, ****p≤0.0001.”

**Reviewer 2**

While vaccine-induced neutralizing antibody responses represent the major correlate of protection against infection and severe disease, there are other antibody-mediated responses that can contribute to protection aside from neutralization. Here, Gorman and colleagues sought to uncover other mechanisms by which vaccines provide protection by dosing down a currently adenoviral platform, Ad26.CoV.2.S, that is the basis of the Johnson and Johnson COVID-19 vaccine. By dosing down the vaccine, they hypothesize they can reveal other important arms of the immune response that are involved in protection, which may often be saturated by current dosing regimens. They found that neutralizing and binding antibody titers were minimally impacted by dosing. However, Fc-effector functions and T cell-mediated immunity were significantly more impacted in the dosed-down group. The authors claim that having robust Fc- and T cell-mediated responses are crucial for controlling viral replication. Overall, the data presented are of sufficient quality in rigor and generally supports the authors' claims. However, the method by which Fc-mediated responses are measured may not truly reflect what may occur during natural infection. This should be addressed by the authors as it has broad implications on how the data concerning the activation of Fc-mediated is interpreted and used as a correlate of protection. There is also some information that may have been unintentionally left out that could further clarify the data for readers. These will be further discussed below.

Strengths:
The experimental design of dosing down a vaccine dose in non-human primates is an excellent approach in revealing arms of the immune response that may or may not be impacted by dosing. One can then correlate specific immunological functions that may be sufficient or deficient with disease phenotype.

Weaknesses:
Based on Figure 1, samples were collected post immunization and challenge (at week 6). However it is unclear if the data presented in Figures 2 to 6 are from which timepoints - are they serum titers after the immunization and at which time point, or are they after the challenge? It was not clearly indicated in the main text or in the figure legend. Please clarify. Also, after clarification, is there a good correlation in the magnitude of the immune response post immunization to post challenge in a dose-dependent manner? This should be further discussed in the text or discussion.

- **Response:** We apologize for the confusion. All system serology samples were generated using samples collected at week 6 post-immunization and pre-challenge. Only pre-challenge serum samples were profiled to examine vaccine induced “correlates” of immunity, similarly to the approach used to define immune correlates of immunity in Phase 3 human trials. Unfortunately, post-infection samples were not collected in this study due to high cost of sampling in the BSL-3 facility early in the pandemic. While this is done far more routinely now, at the time, post-infection samples were not collected at sufficiently frequent timepoints to capture this type of infection. However, we agree that post-challenge profiles could also be quite important, providing critical mechanistic insights into the precise immune functions that are most critical for containment of the virus. These points have been added to the methods and the discussion.

SARS-CoV-2 buds through the ER-Golgi pathway. In fact, the major subcellular localization of the S in the presence of other viral proteins (i.e., M) during natural infection accumulates in the early Golgi (PMID: 25855243, 17166901, 15507643). It is noted that others have used cells stably expressing only the S as target cells, however, it has been demonstrated that the expression of S alone allows for surface expression (PMID: 34504087). It is also noted that a large number of studies have used FluoSphere NeutrAvidin or similar technology use it to couple the spike of a viral protein as a surrogate for phagocytosis assays. While the data presented is of high rigor, it is not known whether the S is sufficiently expressed on the surface of infected cells in nature. At most it may represent how antibodies encounter viral particles, form immune complexes and are taken up by phagocytes through some Fc-mediated mechanism. However, due to how the present authors and others would like to highlight the importance of Fc-mediated immunity in controlling viral replication, one would have to use infected cells as target cells and look for activation of Fc-dependent activation of innate immune cells as an accurate measure. The thinking behind this is that clearance of infected cells requires the target of antibodies be expressed on the surface of infected cells. It would increase confidence in the interpretation of the data if the authors can confirm that indeed the S is the target of Fc-mediated immune response in naturally infected cells - as a mechanism of clearing virally-infected cells.

- **Response:** We thank the reviewer for these critical points, that we agree with. We also do not believe that clearance of “infected cells” occurs during SARS-CoV-2 infection, but instead believe that antibodies play a critical role in rapidly capturing and clearing viral particles. For the reasons raised by the reviewer, related to the limited evidence for expression of Spike on the surface of infected cells (PMID: 27265768), we are unclear what Spike-expression on artificial *in vitro* infected target cells indicates from an immune correlates perspective. Furthermore, early work in natural infection correlates suggested that antibody-mediated NK cell activation was not associated with resolution of severe disease (PMID: 33207184). Conversely, opsonophagocytic functions were tightly linked to protective immunity, further supporting the notion that viral-particle clearance, rather than destruction of infected cells, is likely to be key to protective immunity against this infection. Moreover, given the lower abundance of NK cells in the upper and lower respiratory tract prior to infection, it is unlikely that these cells contribute dominantly to the control/clearance of infection (PMID 33846275 and 32398875). Rather highly abundant resident phagocytic or circulating granulocytes, likely contribute to rapid vaccine mediated capture and elimination of the virus.

However, the reviewer raises an interesting idea related to the generation of an assay that can reliably test the ability of antibodies to drive “infected cell killing”. While several groups have developed surrogate killing assays, they rely on artificial cell types expressing Spike alone, which does not occur in the presence of natural infection, (PMID: 33997824). Moreover, killing assays using authentic epithelial cells has not be performed, largely due to the ability of NK cells to drive heterologous killing of non-HLA matched cells. While we have explored the possibility of doing this in a fully matched rhesus system, capturing rhesus epithelial cells is quite complex, to create an autologous system. Thus, we included our qualified NK cell assay that simply probe the ability to antibodies to leverage NK cell function, in the most agnostic way possible. Thus, while we agree that understanding whether NK cells can kill SARS-CoV-2 infected cells is important, we have found it difficult to set up a “realistic” assay to definitely prove/disprove a role for NK cells in control/clearance of SARS-CoV-2. We have added all these points, as well as the discussion related to the improbable role of NK cell killing of infected cells to the manuscript and agree with the reviewer that these points need to be clearly articulated in our pursuit for a correlate of immunity against SARS-CoV-2.

**Reviewer 3**:

The manuscript submitted by Alter and colleagues uses an innovative dose-down vaccine and challenge model as an approach to define correlates of Ad26.CoV2.S vaccine-mediated protection against SARS-CoV2 in the rhesus macaque model. This work is critically important. Despite success of current vaccine modalities, a clear understanding of correlates of protection remains to be defined. In the current study, the authors demonstrate that neutralizing, binding, and FcR-dependent functional antibody responses were involved in protection, while antibody binding, FcR-dependent functional responses and T cell responses were involved in viral control. Thus, FcR-dependent antibody responses contribute to both protection and control. This suggests a need to evaluate these types of antibody responses against existing and emerging variants to guide vaccine boosting and next-generation vaccine development. The manuscript is well written, well designed, and expected to have broad and substantial impact. There are only a few minor concerns to address.

Lines 105-106 what is meant by "systems serology was applied to the aforementioned BAL and NS samples"? From the methods it would seem that all antibody assays were performed using serum. Please confirm this is the case and clarify in the methods/results. Also, all immune measurements were performed at Week 6 correct? This should also be clearly stated in the methods and the week 2 and 4 collection timepoints should be removed from Figure 1 if they are not relevant to the current study.

- **Response:** We thank the reviewer for pointing out this error. Samples taken from BAL and NS were used only to quantify the level of virus in the system through the use of a subgenomic mRNA assay. The system serology profiling was only conducted on peripheral samples 6 week post-immunization. The lines at week 2 and 4 were removed as suggested.

For ELISPOT assay, were the 200,000 cells/well whole PBMC or T cell enriched?

- **Response:** We thank the reviewer for pointing out this missing detail; the 200,000 cells/well are whole PBMC.

Speculation in discussion on lines 342 and 343 should be removed. The T cell data included does not differentiate between CD8 and CD4, and TFH have not been profiled. Too many assumptions are involved in this statement.

- **Response:** We thank the reviewer for this point and the sentence starting on line 342 and ending on line 343 has been removed entirely.

Can font size on polar plots be increased?

- **Response:** We have adjusted the font size on both polar plots has been increased from 14 to 20.

In the discussion, please reiterate that the results and conclusions are limited to the Ad26-based vaccine. These findings cannot yet be generalized to the other vaccine platforms.

- **Response:** We appreciate the reviewer’s point and have modified the text. Specifically, we have restricted our conclusions to Ad26 vaccine correlates and modified the text to read: “in the context of an Ad26 vaccine” on line 349, and “Whether these findings are generalizable to other vaccine platforms remains to be determined” to the end of the paragraph in line 355.
